# Neurological disorders associated with glutamic acid decarboxylase 65 antibodies: Clinical spectrum and prognosis of a cohort from China

**DOI:** 10.3389/fneur.2022.990553

**Published:** 2022-10-05

**Authors:** Lin Bai, Haitao Ren, Menglin Liang, Qiang Lu, Nan Lin, Mange Liu, Siyuan Fan, Ruixue Cui, Hongzhi Guan

**Affiliations:** ^1^Department of Neurology, Peking Union Medical College Hospital, Peking Union Medical College and Chinese Academy of Medical Sciences, Beijing, China; ^2^Department of Nuclear Medicine, Peking Union Medical College Hospital, Peking Union Medical College and Chinese Academy of Medical Sciences, Beijing, China

**Keywords:** encephalitis, autoimmune disease, glutamic acid decarboxylases 65, antibody, immunotherapy

## Abstract

**Objective:**

To describe clinical phenotypes and prognosis of neurological autoimmunity related to glutamic acid decarboxylase 65 (GAD65) antibodies in China.

**Method:**

In this retrospective observational study from Peking Union Medical College Hospital, we identified patients with neurological disorders related to GAD65 antibodies (cell-based assay) from May 2015 to September 2021. Clinical manifestations, immunotherapy responsiveness, and outcomes were collected after obtaining informed consent from all patients.

**Results:**

Fifty-five patients were included: 40 (72.73%) were women and initial neurological symptoms developed at 42(34-55) years of age. The median time to the nadir of the disease was 5 months (range from 1 day to 48 months). The clinical syndromes included limbic encephalitis (LE) or epilepsy (Ep) (*n* = 34, 61.82%), stiff-person syndromes (SPS) (*n* = 18, 32.73%), autoimmune cerebellar ataxia (ACA) (*n* = 11, 20%), and overlap syndrome in eight (14.55%) patients. Thirty-two (58.2%) patients had comorbidities of other autoimmune diseases, including Hashimoto thyroiditis (*n* = 17, 53.13%), T1DM (*n* = 11, 34.78%), vitiligo (*n* = 6, 18.75%), and others (n=5, 15.63%). Two (3.64%) patients had tumors, including thymoma and small cell lung cancer. Fifty-one (92.7%) patients received first-line immunotherapy (glucocorticoids and/or IV immunoglobulin), and 4 (7.3%) received second-line immunotherapy (rituximab). Long-term immunotherapy (mycophenolate mofetil) was administered to 23 (41.8%) patients. At the median time of 15 months (IQR 6–33.75 month, range 3–96 month) of follow-up, the patients' median modified Rankin Score (mRS) had declined from 2 to 1. Thirty-eight (70.4%) patients experienced clinical improvement (mRS declined ≥1), 47 (87%) had favorable clinical outcomes (mRS ≤2), and nine were symptom-free (16.7%). The sustained response to immunotherapy ranged from 7/15 (63.63%) in ACA patients and 22/34 (64.7%) in LE/Ep patients to 14/17 (82.35%) in SPS patients.

**Conclusions:**

LE/Ep was the most common neurological phenotype of GAD65 antibody neurological autoimmunity in our cohort. Most patients had comorbidities of other autoimmune diseases, but underlying tumors were rare. Most patients responded to immunotherapy. However, the long-term prognosis varied among different clinical phenotypes.

## Introduction

Glutamic acid decarboxylase (GAD) is a rate-limiting enzyme in the synthesis of the inhibitory neurotransmitter gamma-aminobutyric acid (GABA). It consists of two isoforms — GAD65 and GAD67. GAD65 is highly enriched in nerve terminals (1) and mediates activity-dependent GABA synthesis when postsynaptic inhibition is needed (2). While GAD67 produces foundational neuronal cytosolic GABA (3). Autoantibodies against GAD may disrupt the synthesis of GABA and impair GABAergic inhibitory circuits.

GAD65 antibodies are associated with diabetes mellitus type 1 (T1DM) and diverse neurologic disorders. They were initially characterized in a patient with stiff-person syndrome (SPS) and T1DM in 1988 (4). Subsequently, GAD65 antibodies were also identified in patients with autoimmune cerebellar ataxia (ACA), limbic encephalitis (LE) and epilepsy (Ep).The complexity of the disease is influenced by diverse clinical phenomena and different prognoses. It is challenging for physicians to diagnose and treat. Recently, Muñoz-Lopetegi et al. (5) and Budhram et al. (6) reported case series of Caucasian patients. However, few large cohorts of GAD65 antibodies associated disorders have been reported in East Asia (7). In this study, we reported a case series in China to offer further insights into the clinical phenotypes and prognosis of GAD65 antibodies associated disorders.

## Methods

### Patients

Patients with GAD65 antibodies and neurologic symptoms (encephalopathy, epilepsy, psychiatric symptoms, rigidity, movement disorders, gait disturbances, diplopia, and sleep disorders) were enrolled between May 2015 and September 2021 in Peking Union Medical College Hospital (PUMCH) Encephalitis and Paraneoplastic Syndrome Project. GAD65 antibodies were detected by a cell-based assay (CBA). Clinical information was obtained from the patients' medical files. The data included age, gender, CSF test, MRI, EEG, therapeutic regimens, and treatment outcomes.

### Standard protocol approvals, registrations, and patient consent

The institutional review board of PUMCH approved the study protocol (JS-891). Written informed consent was obtained from all patients.

### Definition of the clinical phenotypes, immunotherapy regimen, and follow-up

LE was identified as subacute onset (rapid progression of fewer than 3 months) of working memory deficits, seizures, or psychiatric symptoms with medial temporal lobe T2-hyperintensity. Ep was classified by the International League Against Epilepsy 2017 (8). Overlap syndromes were identified when patients present with more than one neurologic syndrome. LE with Ep alone was not classified as an overlap syndrome.

The immunotherapy responses were assessed from the medical files. Clinical improvement was defined as a decrease in the modified Rankin score (mRS) (≥1 point) from that at the previous visit. For patients with Ep, at least 50% seizure frequency reduction was considered an improvement. A favorable outcome was defined as an mRS ≤2, and a poor outcome was defined as an mRS >2 at the end of follow-up.

### Laboratory tests

The cerebrospinal fluid (CSF) and serum samples were tested using a CBA (EUROIMMUN, Lübeck, Germany; REF: FA 1022-1005-50) in the neurological immunology laboratory of PUMCH. The antibody titers were measured using serial dilutions of serum and CSF until the reactivity was no longer visible.

### Statistics

The median with the interquartile range (IQR) was used in continuous variables. Categorical variables are reported as numbers (percentages). We used Pearson's χ^2^ or Fisher's exact test for multiple categories and a *t*-test or the Mann–Whitney U test for continuous variables. A two-sided *p* < 0.05 was considered statistically significant. We used SPSS 24.0 for analysis.

## Results

### Clinical characteristics and syndromes

**Figure 1:**
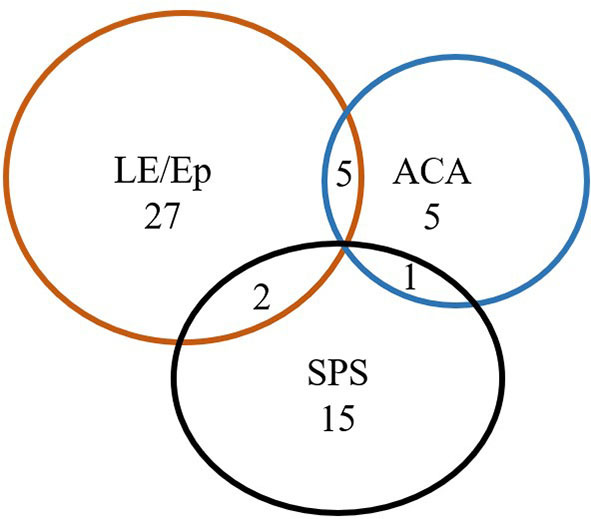
Neurological autoimmunity associated with anti-GAD65 antibodies. LE, limbic encephalitis; Ep, epilepsy; SPS, stiff person syndromes; ACA, autoimmune cerebellar ataxia.

Fifty-five patients were enrolled. The median age at onset was 42(34-55) years of age. Forty (72.7%) patients were females. The median time nadir was 5 months (range 1 day-6 months). Most common clinical manifestations were seizures (*n* = 30, 54.5%), progressive proximal limb rigidity (*n* = 18, 32.7%), working memory deficits (*n* = 17, 31.9%), and ataxia (*n* = 11, 20%). One patient manifested rapid-eye-movement sleep behavior disorder (RBD). All patients had typical neurological syndromes related to GAD65 antibody: Ep (*n* = 8, 14.5%), LE (*n* = 26, 47.3%), SPS (*n* = 18, 32.7%), and ACA (*n* = 11, 20%), and 8 (14.5%) patients had overlap syndromes (Figure 1, Table 1).

**Table 1:** Characteristics of the 55 patients with GAD65-ab associated with neurological autoimmunity.

**Characteristics**	**Values**
Female sex, *n* (%)	40 (72.73%)
Age, y (IQR)	42 (34-55)
Time to nadir, median (IQR; range)	5 months (1 d-6months; 1 d-48month)
**Clinical symptoms**, ***n*** **(%)**	
Seizures	30/55 (54.5%)
Muscle rigidity	18/55 (32.7%)
Memory deficits	17/55 (31.9%)
Ataxia	11/55 (20%)
Diplopia	7/55 (12.7%)
Psychosis	6/55 (10.9%)
Dysarthria	4/55 (7.3%)
RBD	1/55 (1.8%)
Autoimmune Comorbidities *n* (%)	32/55 (58.18%)
Hashimoto thyroiditis	17/32 (53.12%)
T1DM	11/32 (34.38%)
Vitiligo	6/32 (18.75%)
Rheumatoid arthritis	1/32 (3.13%)
Grave‘s disease	1/32 (3.13%)
Psoriasis	1/32 (3.13%)
Thrombocytopenia	1/32 (3.13%)
Myasthenia gravis	1/32 (3.13%)
Tumor found within 5 years of symptom onset	2/55 (3.34%)
Thymoma	1/2 (50%)
Small cell lung cancer	1/2 (50%)

RBD, rapid-eye-movement sleep behavior disorder; T1DM, type 1 diabetes mellitus; Time to nadir. The time from symptoms onset to attend a hospital.

#### LE and Ep

Thirty-four patients were involved, with 23 (67.6%) females. The patients had either an acute or a subacute onset (2 months vs. 10 months, *P* = 0.001). Fifty patients (91%) presented with tonic-clonic epileptic seizures, and all had focal onset, including aware motor onset (7/34, 23.3%) (such as lip-smacking, wandering, or other automatic activities), non-motor onset (18/34, 60%) (such as emotional seizures and sensory seizures), or impaired awareness (5/34, 16.7%). Seventeen (31.9%) patients were disturbed by memory decline: 10 of them acutely started, and seven patients had symptoms that occurred approximately half a year later.

#### SPS

Eighteen patients were involved, with 14 (77.8%) females. The median duration was 13 months, which was longer than the other phenotypes (13 m vs. 3 m, *P* = 0.000). The majority of the patients (15/18, 83.3%) had symptoms that began from the lower back or bilateral proximal limb. One patient had symptoms that began from the left lower limb (1/18, 5.56%), and two patients began from the neck and shoulders (2/18, 11.1%). Four patients manifested mild upper motor neuron (UMN) signs (brisk reflexes and Babinski sign).

#### ACA

There were 11 patients with a median onset age of 54 years, which was older than the patients with other GAD65 antibody related neurologic disorders (54 years vs. 41 years, *p* = 0.039). Gait ataxia was most frequently documented (11/11, 100%), followed by dizziness or diplopia (7/11, 63.6%), and dysarthria (4/11, 63.4%) (Table 2).

**Table 2:** Different clinical syndromes of GAD65-ab-associated neurological autoimmunity.

**Clinical syndrome**	**LE/Ep (*N* = 34)**	**SPS (*N* = 18)**	**ACA (*N* = 11)**
Age, y, median (IQR, range)	41 (31–55.5, 6–75)	43( 37-49,13-62)	54 (43–63, 29–75)
Female sex, n (%)	23 (67.6%)	14 (77.8%)	9 (81.8%)
Time to nadir, month, median (IQR; range)	2 (0.34–6, 0.03-48)	13 (3.75–48, 1–48)	6 (2–6, 0.03–15)
Autoimmune Comorbidities, *n* (%)	19 (55.9%)	11 (61.1%)	4 (36.4%)
Oncology	0	2	1
CSF WBC>5 × 106/L	3/22 (13.6%)	3/13 (23.1%)	2/9 (22.2%)
CSF OCB (+)	10/15 (66.7%)	9/10 (90%)	4/6 (66.7%)
Favorable outcome	22/34 (64.7%)	16/17 (94.1%)	7/11 (63.6%)

CSF, cerebrospinal fluid; OCB, oligoclonal bands; LE, limbic encephalitis; Ep, epilepsy; SPS, stiff person syndromes; ACA, autoimmune cerebellar ataxia; Time to nadir, The time from symptoms onset to attend a hospital; Favorable outcome, A favorable outcome was defined as an mRS ≤ 2 at the end of follow-up.

#### Overlap

Eight patients had combined syndromes, including LE/Ep combined with CA (*n* = 5, 62.5%), LE/Ep plus SPS (*n* = 2, 25%), and SPS plus CA (*n* = 1, 12.5%), with an interval mediation time of 10.5 months (4.5–22.5). LE/Ep was most likely to merge with other syndromes. Details were provided in the supplementary materials (Supplementary Table 1).

#### Oncology

Two (3.6%) female patients had a tumor within 5 years of symptom onset. One patient with SPS was diagnosed with thymoma (B1 type) at the onset, and her symptoms progressed after the thymoma removal. The other patient with small cell lung cancer (cTxN1M0, IIa) felt neck and shoulder stiffness at the onset and began to have diplopia and stumbling 6 months later. She received antitumor therapy and IVIG treatment. Neck and Shoulder stiffness disappeared, but ataxia was not approved (mRS 4).

### Ancillary test results

#### Neuroimage

All patients underwent brain magnetic resonance imaging (MRI), and 36.36% (20/55) of the patients showed abnormal results, including 16 medial temporal lobe abnormalities, two multifocal cortical lesions, and two mild cerebellar atrophy.

**Figure 2:**
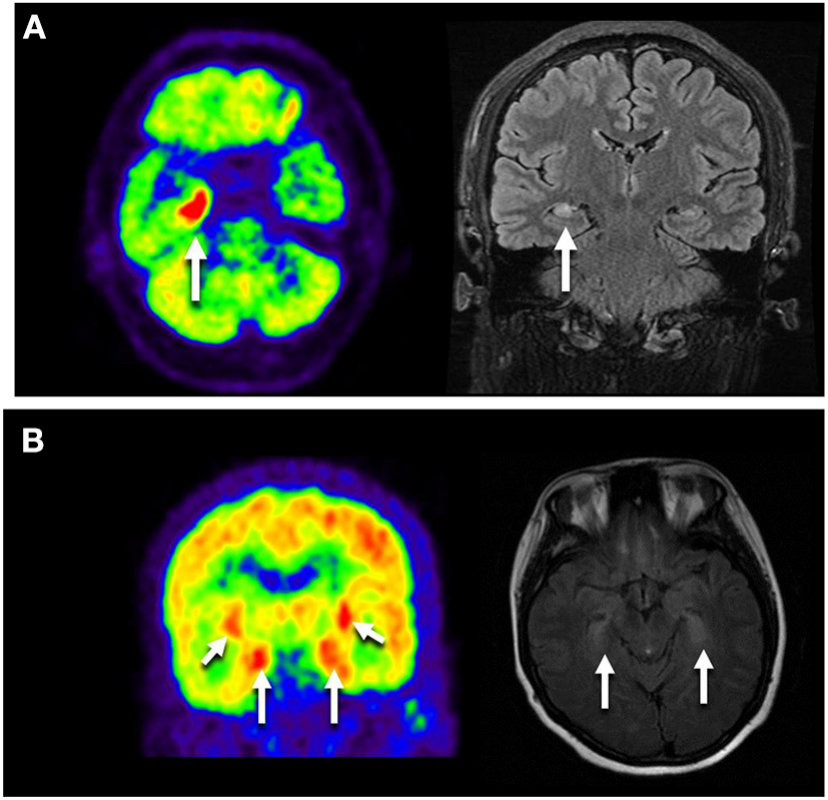
Neuroimages of the GAD65 antibody associated neurological disorders. **(A)** Images of NO.9 who presented with Ep. Axial FLAIR MRI showed right temporal hyperintensity, and Axial FDG-PET/CT scan showed hypermetabolic right mesiotemporal spot (white arrow). **(B)** Images of NO.41 who manifested as LE plus ACA. 18F-FDG PET/CT showed increased FDG uptake in the bilateral medial temporal lobes. MRI showed bilateral medial temporal lobe T2 hypertension. LE, limbic encephalitis; Ep, epilepsy; ACA, autoimmune cerebellar ataxia.

Thirteen patients completed 18F-FDG PET/CT scans: one presented with multicortical hypermetabolism, four with temporal lobe hypermetabolism, and one with cerebellar hypometabolism (Figure 2).

#### Electrophysiology

EEG was available for patients with LE/Ep. There were 16 patients with abnormal EEG findings, showing abnormal discharges in the temporal lobe. Ten patients with SPS underwent multichannel surface electromyography, and 9/10 showed continuous motor unit activity in at least one axial muscle. One was normal because of clonazepam usage.

#### CSF

CSF white blood cell (WBC) count data were available for 37 patients. The median WBC count was 1 (IQR 0–4) × 10^6^/L. Seven (18.9%) patients had pleocytosis. Twenty-six patients underwent CSF oligoclonal immunoglobulin G (IgG) bands (OCB) test. Seven patients had no oligoclonal bands despite having positive GAD65 antibodies in the CSF.

### Treatment outcomes

#### Therapy

Fifty-one (92.7%) patients received immunotherapy. Forty-two (77.8%) patients received intravenous immunoglobulins (IVIG) (0.4 g/kg for 5 days), and 44 patients (81.5%) received intravenous methylprednisolone (IVMP). Four patients additionally received rituximab for second-line immunotherapy. Long-term immunotherapy with Mycophenolate Mofetil (MMF) was given in 23 (41.8%) patients as a maintenance therapy to prevent and manage relapses. Four patients refused immunotherapy, including two with SPS and two with Ep. They received treatment with clonazepam or antiepileptic drugs because of mild clinical symptoms.

#### Outcome

The median follow-up time was 15 months (IQR 6–33.75month, range 3–96 month). One patient with tumor-negative SPS failed to follow up. Thirty-eight (70.4%) patients experienced clinical improvement, and 9 (16.7%) were symptom-free. Forty-seven (87%) patients attained satisfactory neurologic function (mRS 0–2). The median mRS declined from 2 (IQR 2–3) to 1 (IQR 1–2). There were no deaths in our group, but one patient failed to attend the follow-up.

**Figure 3:**
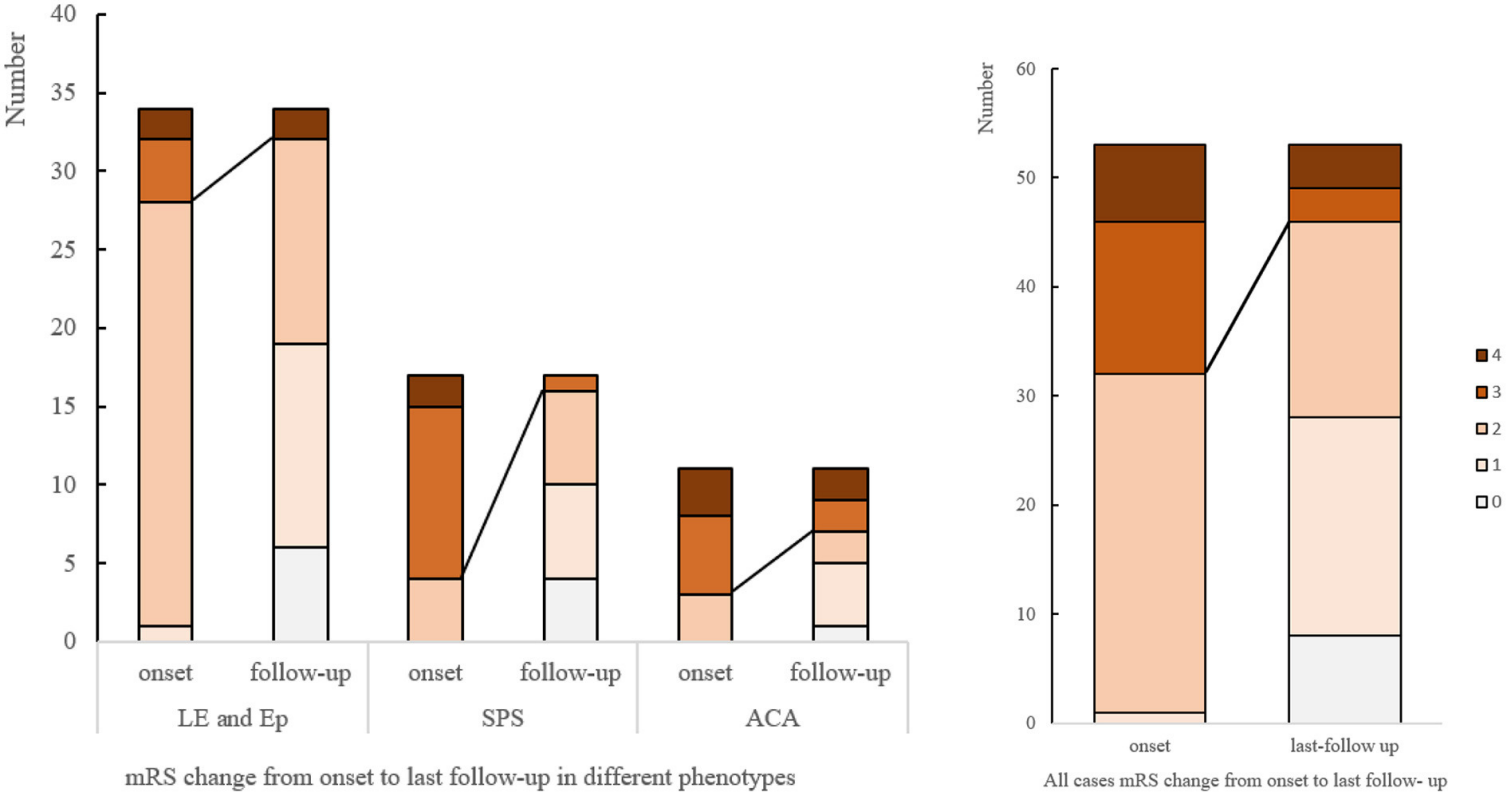
mRS changes from onset to last follow-up. mRS, modified Rankin score; LE, limbic encephalitis; Ep, epilepsy; SPS, stiff person syndromes; ACA, autoimmune cerebellar ataxia.

The median mRS in patients with SPS dropped from 3 (IQR 2.5-3) to 1 (IQR 0.5–2), with 14 (82.35%) patients showing clinical improvement, and 4 (22.2%) completely recovered. For CA, the median mRS score declined from 3 (IQR 2–4) to 2 (IQR 1–3). For LE or Ep patients, the median mRS changed from 2 (IQR 2-2) to 1 (IQR 1–2). 22 (64.7%) patients achieved a >50% seizure frequency reduction, and 5 (14.7%) were symptom-free. Sustained response to immunotherapy ranged from 63.6% in ACA and 64.7% in LE/Ep to 82.35% in SPS (Figure 3).

Most patients with LE converted to chronic epilepsy during the follow-up. The patients with LE or Ep had the same median mRS change after treatment, from two (IQR 2-2) to 1 (IQR 1–2). There was no statistically significant difference in the prognosis between the two groups (*P* = 0.681) when a clinical improvement was defined as a seizure frequency reduction ≥50% (Table 3).

**Table 3:** Comparisons of the clinical data of the patients with GAD65-ab-associated neurological disorders.

	**Favorable outcome**	**Poor outcome**	***P*-value**
Age (y)	42.5(12-71)	42.5(6-75)	0.766
**Sex (** ***n*** **, %)**			
Male	11/15	4/15	1.00
Female	27/39	12/39	
Diagnosis duration (month)	3 (0.03–48)	6.5 (0.03–21)	0.371
**Symptoms (** ***n*** **, %)**			
SPS	14/17	3/17	0.191
LE/Ep	22	12	0.279
ACA	7	4	0.716
**Immunotherapy (** ***n*** **, %)**			
First-line	18/27	9/27	0.551
First-line + MMF	15/19	4/19	0.309
Second line	1/4	3/4	0.073
Baseline mRS score >2 (*n*, %)	17/22	5/22	0.357

LE, limbic encephalitis; Ep, epilepsy; SPS, stiff person syndromes; ACA, autoimmune cerebellar ataxia; mRS, modified Rankin Score; MMF mycophenolate mofetil; IVIG, Intravenous Immunoglobulin; MP, methylprednisolone; First-line, IVIG and IVMP alone or combine; Second line, Rituximab.

Twenty-eight (50.9%) serum samples were available before and after the treatment. Seventeen (60.7%) patients' serum GAD65-ab titers were unchanged, while 13/28 (46.4%) had decreased. GAD65-ab did not turn seronegative. There was no significant correlation between the serum GAD65 antibody titer variation and prognosis (*P* = 0.671).

## Discussion

This study aims to describe the clinical characteristics and prognosis of GAD65 antibodies related neurological disorders. To the best of our knowledge, this is the largest single-center cohort from East Asia. Our study provides several relevant findings: (1) The disorder predominately involves middle-aged females. (2) Patients present with several specific neuroimmune phenotypes, including CA, SPS, LE, and Ep. Patients with LE tend to get chronic seizures. (3) Most patients have autoimmune comorbidities, but underlying tumors are unusual. (4) Most patients experienced clinical improvement after immunotherapy, but only a minority remain symptom-free.

GAD65 antibodies related neurological disorders present with a set of well-established symptoms, including SPS, ACA, LE, and Ep. SPS usually involves the axial muscles and proximal limb muscles. Some patients have UMN manifestations that support spinal cord involvement (6, 9). In patients with epilepsy, abnormal discharges usually originate from the temporal lobe (10–12). We found that patients with LE can present with chronic seizures during years of follow-up and there was no significant difference in prognosis between LE and Ep (*P* = 0.681). This finding may indicate that the two clinical phenotypes have some common clinical features and underlying pathophysiology.

Autoimmune comorbidities are frequent in patients with GAD65-ab related neurological disorders, including autoimmune thyroid disease (30–48%), T1DM (11–30%), vitiligo (2–16%), and rheumatic disorders (6–7%) (10, 13–15). GAD antibodies were positive in over 50 % of patients with T1DM, and these patients had a higher prevalence of autoimmune thyroiditis than anti-GAD-negative patients with T1DM (16). Besides, A previous study has indicated that vitiligo may be a diagnostic clue to an autoimmune cause of encephalitis (17). The autoantibodies induced by the exposure antigen triggered by vitiligo are more likely to attack the mimic extracellular epitopes of neurons because both the skin and the nervous system are derived from the external germ layer. Further studies are needed to investigate the relationship between these diseases.

Approximately 4–11% of patients have underlying tumors (5–7, 18). In our group, 3.64% (*n* = 2) of the patients had manifestations of atypical SPS syndrome and overlap (SPS+CA) syndrome combined with tumors. This finding is lower than that of a previous study and suggests that patients presenting with CA, SPS, or atypical syndromes should be vigilant.

CSF tests and neuroimages are important for disease diagnosis. The CSF cell counts and protein levels are usually normal, but 40–70% of patients have oligoclonal immunoglobulin G (IgG) bands (19). Brain MRI shows parenchymal atrophy, cortical/subcortical parenchymal T2 hyperintensity, and abnormal hippocampal signals in LE/Ep (20). 18F-FDG PET/CT shows FDG uptake in the parietotemporal lobes (21). For patients with chronic epilepsy, hypometabolism in the mesial temporal lobe areas, together with hypometabolism in the insulae and medial inferior frontal-hypothalamus, may be characteristic of patients with GAD65-ab (22).

Immunotherapy is the main treatment strategy for anti-GAD65-related neurological disorders (23). However, there is a lack of consensus on the immunotherapy regimen. A study showed that IVIG had a better therapeutic effect than IVMP for patients with SPS and ACA (24). Another study found that corticosteroids were the best regimen for ACA (25). Some research indicates that there was no significant difference in the effectiveness between the two regimens (26, 27). Studies found that rituximab lacked efficacy in patients with SPS (28, 29). However, there were opposite standpoints reported (30, 31). Researchers found that tocilizumab was helpful for super-refractory status epilepticus (32). There are possible benefits from epilepsy surgery in some anti-GAD65-LE (14). Saidha et al. reported that MMF was effective in the treatment of anti-GAD65-associated limbic encephalitis (9, 33). Clinical treatment should take the disease severity, comorbidities, and patient economic situation into account to make an appropriate strategy.

Most patients show clinical improvement after immunotherapy. Previous studies reported that 63.6%-95% of patients experience clinical improvement, but only 0–1% of patients who present with ACA or Ep are symptom-free (6). This indicates that anti-GAD65-related neurological autoimmunity is a chronic disease. SPS has a better response to immunotherapy than epilepsy and ACA (34–36). The different prognoses may indicate that the target antigens are different among clinical phenotypes. In patients with pre-and post-treatment samples, serum GAD-Ab titers became lower after initial improvement and unchanged during follow-up (5, 37). In our cohort, most patients had satisfactory neurologic function (mRS≤2), but complete recovery only occurred in a minority of patients. About 40% of patients received MMF as long-term immunotherapy in this study. We found that MMF (*p* = 0.306) did not improve the clinical outcomes, possibly because of the relatively small sample size, and the role of long-term immunotherapy needs further investigation.

There are limitations to this study. (1) As the national referral center for complicated diseases, our cohort may be biased by more complicated and refractory cases, which may introduce bias in the characterization of the entity we aim to describe in this review. (2) We collected clinical data from medical records, which may lead to an overestimation of some symptoms. (3) We need to find more suitable evaluation plans for each syndrome rather than mRS. (4) This study is a single-center cohort. More accurate prognostic evaluation warrants further large-size cohort investigation and extended follow-up.

To summarize, GAD65 antibody related neurological disorders present with various clinical symptoms, and LE/Ep are the most common phenotypes in Chinese patients. The majority of patients have clinical improvement after immunotherapy, but full recovery only occurs in a small proportion of patients. Therefore, this condition seems to be a chronic disease. Multicenter, large-cohort studies are needed to reach a consensus for standardizing the immunotherapy regimens and to obtain further insights into the prognosis.

## Data availability statement

The original contributions presented in the study are included in the article/Supplementary material, further inquiries can be directed to the corresponding author/s.

## Author contributions

LB: data curation, writing the original draft, and submitting. HR: data collection and data analysis. ML: writing the orginal draft and supervision. SF and RC: supervision. HG: supervision, writing-reviewing, and editing. QL and NL: article supervision in the process of manuscript revision. All authors agree to be accountable for the content of the work. All authors contributed to the article and approved the submitted version.

## Funding

CAMS Innovation Fund for Medical Sciences (CIFMS 2021-I2M-1-003).

## Conflict of interest

The authors declare that the research was conducted in the absence of any commercial or financial relationships that could be construed as a potential conflict of interest.

## Publisher's note

All claims expressed in this article are solely those of the authors and do not necessarily represent those of their affiliated organizations, or those of the publisher, the editors and the reviewers. Any product that may be evaluated in this article, or claim that may be made by its manufacturer, is not guaranteed or endorsed by the publisher.
