# Supplementary material for: Neurological disorders associated with glutamic acid decarboxylase 65 antibodies: Clinical spectrum and prognosis of a cohort from China
Source: Front Neurol. 2022 Oct 5;13:990553. doi: 10.3389/fneur.2022.990553 (PMC9581312; doi:10.3389/fneur.2022.990553)
Supplement: Supplementary file 1 [file Table_1.docx]

**sTable 1** the clinical characteristics of patients with overlap syndrome in detail.

| ID | condition | onset | best after treatment | therapy | mRS score  (before/after) |
| --- | --- | --- | --- | --- | --- |
| 1 | LE/EP + ACA | seizures, memory impairment.  walked tandem impossible (16m later). | seizure frequency unchanged.  ataxia improved. | IVIG, MP, MMF | 3/2 |
| 2 | Ep + SPS | seizures.  trunk and limb stiffness, walk with assistant (1 month later). | seizure redaction＞50%.  stiffness improvement. | IVIG, MP, MMF | 3/1 |
| 3 | LE/EP+ACA | memory impairment, psychosis.  ataxia, not able to walk (14m later). | no improvement | MP | 4/4 |
| 4 | ACA +Ep | dizziness, [blurred vision](http://www.youdao.com/w/blurred%20vision/#keyfrom=E2Ctranslation), walking instability. Seizures (7month later) | seizure redaction＜50%.  ataxia improved. | IVIG, MP | 3/2 |
| 5 | ACA +Ep | moderate ataxia.  Seizures (5y later) | seizure frequency unchanged.  ataxia improved. | MP, IVIG | 2/1 |
| 6 | EP + ACA | seizures and memory impairment. Walked tandem impossible (4m later) | seizure frequency unchanged. ataxia improved. | IVIG, MP, MMF | 2/1 |
| 7 | ACA+SPS | dizziness, walking instability.  neck and limb stiffness (6month later) | Stiffness improved.  but ataxia unchanged | IVIG, MP | 4/4 |
| 8 | SPS+LE/EP | memory impairment.  lower back and limb stiffness. | Not improved | IVIG, MP, MMF, RTX | 2/2 |

*IVIG: [Intravenous Immunoglobulin](https://www.abbreviationfinder.org/cn/acronyms/ivig_intravenous-immunoglobulin.html#:~:text=IVIG%20%E4%BB%A3%E8%A1%A8%20%E9%9D%99%E8%84%89%E6%B3%A8%E5%B0%84%E5%85%8D%E7%96%AB%E7%90%83%E8%9B%8B%E7%99%BD%E3%80%82%20%E5%A6%82%E6%9E%9C%E6%82%A8%E6%AD%A3%E5%9C%A8%E8%AE%BF%E9%97%AE%E6%88%91%E4%BB%AC%E7%9A%84%E9%9D%9E%E8%8B%B1%E8%AF%AD%E7%89%88%E6%9C%AC%EF%BC%8C%E5%B9%B6%E5%B8%8C%E6%9C%9B%E7%9C%8B%E5%88%B0,%E9%9D%99%E8%84%89%E6%B3%A8%E5%B0%84%E5%85%8D%E7%96%AB%E7%90%83%E8%9B%8B%E7%99%BD%20%E7%9A%84%E8%8B%B1%E6%96%87%E7%89%88%E6%9C%AC%EF%BC%8C%E8%AF%B7%E5%90%91%E4%B8%8B%E6%BB%9A%E5%8A%A8%E5%88%B0%E5%BA%95%E9%83%A8%EF%BC%8C%E6%82%A8%E5%B0%86%E7%9C%8B%E5%88%B0%20%E9%9D%99%E8%84%89%E6%B3%A8%E5%B0%84%E5%85%8D%E7%96%AB%E7%90%83%E8%9B%8B%E7%99%BD%20%E5%9C%A8%E8%8B%B1%E8%AF%AD%E4%B8%AD%E7%9A%84%E5%90%AB%E4%B9%89%E3%80%82), MP: methylprednisolone, MMF: [Mycophenolate Mofetil](http://www.youdao.com/w/Mycophenolate%20Mofetil/#keyfrom=E2Ctranslation), RTX rituximab.
